# Calculation of Some Low-Lying Electronic Excitations of Barium Monofluoride Using the Equation of Motion (EOM)-CC3 Method with an Effective Core Potential Approach

**DOI:** 10.3390/molecules29184356

**Published:** 2024-09-13

**Authors:** Marko Horbatsch

**Affiliations:** Department of Physics and Astronomy, York University, Toronto, ON M3J 1P3, Canada; marko@yorku.ca

**Keywords:** molecular structure, electronic excitations, coupled-cluster methods

## Abstract

Barium monofluoride (BaF) is a polar molecule of interest in measurements of the electron electric dipole moment. For this purpose, efforts are underway to investigate this molecule embedded within cryogenic matrices, e.g., in solid Ne. For a theoretical understanding of the electronic structure of such an embedded molecule, the need arises for efficient methods which are accurate but also able to handle a number of atoms which surround the molecule. The calculation for gas-phase BaF can be reduced to involve only outer electrons by representing the inner core of Ba with a pseudopotential, while carrying out a non-relativistic calculation with an appropriate basis set. Thus, the method is effectively at a scalar-relativistic level. In this work, we demonstrate to which extent this can be achieved using coupled-cluster methods to deal with electron correlation. As a test case, the SrF($X^{\;2}\Sigma^{+}\to B^{\;2}\Sigma^{+}$) transition is investigated, and excellent accuracy is obtained with the EOM-CC3 method. For the BaF($X^{\;2}\Sigma^{+}\to {A^{\prime }}^{\;2}\Delta$, $X^{\;2}\Sigma^{+}\to A^{\;2}\Pi$, $X^{\;2}\Sigma^{+}\to B^{\;2}\Sigma^{+}$) transitions, various coupled-cluster approaches are compared with very good agreement for EOM-CC3 with experimentally derived spectroscopic parameters, at the level of tens of ${\mathrm{cm}}^{-1}$. An exception is the excitation to the ${A^{\prime }}^{\;2}\Delta$ state, for which the energy is overestimated by $230\;{\mathrm{cm}}^{-1}$. The poor convergence behavior for this particular state is demonstrated by providing results from calculations with basis sets of n = 3, 4, 5)-zeta quality. The calculated excitation energy for the $B^{\;2}\Sigma^{+}$ state agrees better with a deperturbation analysis than with the effective spectroscopic value, with a difference of $120\;{\mathrm{cm}}^{-1}$.

## 1. Introduction

The search for the electron’s electric dipole moment [1,2], as well as laser cooling with the goal of optical trapping, has triggered renewed interest in polar diatomic molecules, such as barium and strontium monofluoride (BaF and SrF). On the theoretical side, this interest is reflected in large-scale computational efforts: a comprehensive study of the low-lying excitations was performed using a relativistic Fock-space coupled cluster (CC) approach [3]. These calculations led to a much improved agreement with experimental data, in particular for the excitation energies $\Delta T_{e}$, i.e., the differences in potential energy curves at the respective minima. Agreement was found at the level of $100\;{\mathrm{cm}}^{-1}$ for SrF and BaF (and larger for CaF), which represents a dramatic improvement over the previous theoretical results. Subsequently, a fully relativistic treatment, including quantum electrodynamic corrections and basis set extrapolation, led to an order-of-magnitude improvement for BaF [4], and agreement with experimental excitation energies $T_{e}$ at the level of $10\;{\mathrm{cm}}^{-1}$. The all-electron Fock space CC method has been shown recently to account for a number of properties, such as ionization energies [5], and hyperfine constants [6].

Since all-electron calculations are computationally challenging, particularly in the relativistic frameworks, it is of interest to explore whether effective core potentials (ECPs) and corresponding basis sets can be combined with high-order CC methods to reach the $100\;{\mathrm{cm}}^{-1}$ level precision with reduced computational effort. Our interest in this regard is the investigation of these molecules within a cryogenic matrix environment, such as argon or neon [7,8,9]. To investigate BaF in a such an environment, a more economical approach is required, and this represents the motivation for this study. The present work deals only with gas-phase molecules and is much simpler than what needs to be performed for molecules embedded in a crystal [10]. A study which demonstrates recent advances in dealing with the effect of phonon interactions on excited electronic energy levels can be found in Ref. [11].

The layout of the paper is as follows. We begin in Section 2 with a short summary of the basic equations which lead to the spectroscopic constants. In Section 3, we demonstrate how well the Equation of Motion (EOM)-CC3 method combined with an ECP and appropriate basis set works for the $X^{\;2}\Sigma^{+}\to B^{\;2}\Sigma^{+}$ transition in SrF, which is free of spin–orbit interactions. We then apply the EOM-CC3, EOM-CCSD, and state-specific Δ-CC methods to the low-lying excitations in BaF. We draw some conclusions in Section 4.

## 2. Methodology

There are a number of coupled-cluster (CC) methods to compute electronic excitations [12,13]. We recently compared some of them for magnesium monofluoride and found that the EOM-CC3 method, when combined with the aug-cc-pVnZ basis set family, can reach agreement with experimentally derived electronic excitation energies $T_{e}$ [14]. The goal of the present work is to show that this methodology can work equally well for heavier systems (such as SrF or BaF) when reducing the all-electron problem to a (10+9)-electron problem by means of an ECP [15,16]. We use the correlation consistent basis sets developed by Hill and Peterson [17].

To achieve high accuracy for the electronic excitation energies of molecules, the following two methods are employed: EOM-CC3 with two basis set families, cc-pVnZ-PP and the augmented version aug-cc-pVnZ-PP. For the former basis set family, we are able to also apply the state-specific Δ-CC methods which were reviewed in Ref. [18]. These methods can be computationally economical: one generates an unrestricted Hartree–Fock (HF) Slater determinant after orbital rotation (switching the highest occupied and some low unoccupied orbitals) to obtain a Δ-SCF solution; then, one follows up with a CC calculation for this state. The problem with this method is that for some large basis sets, the HF calculation collapses back to the ground state. The computations in the Δ-CC step for the excited state usually require many more iterations than for the ground state. An interesting feature, however, is the option to treat triple excitations perturbatively, i.e., there is a Δ-CCSD(T) method for which there is no EOM counterpart.

One question we are interested in is how big the differences are between the state-specific vs. EOM methods, while using the cc-pVnZ-PP basis at the n=5 (5Z) level. The calculations are performed with the CCEOM package within Psi4 [19]. It allows for orbital rotations in the first irreducible representation, and we are able to carry out the necessary rotations by using different symmetries: $C_{2v}$, $C_{s}$, and $C_{1}$. Our focus is on the BaF excitations from the $X^{\;2}\Sigma^{+}$ to ${A^{\prime }}^{\;2}\Delta$, $A^{\;2}\Pi$, and $B^{\;2}\Sigma^{+}$ states.

To obtain the spectroscopic parameters, one has to calculate potential energy curves over some range of molecular separations $R_{i}$. A simpler task is to choose a fixed separation $R=R_{e}$ (e.g., the $X^{\;2}\Sigma^{+}$ value of $R_{e}$ known from experiments), and to compute vertical excitation energies ΔT. The ΔT values overestimate the excitation energies $\Delta T_{e}$, which are obtained from the potential energy curves for both ground and excited states using the respective state-specific equilibrium separations $R_{e}$.

The potential energy curves lead to the spectroscopic parameters $\omega_{e}$ and $\omega_{e}x_{e}$, which follow from the nuclear vibrational excitation energies in accord with [20]

(1) $$E_{v}=T_{e}+\omega_{e}\left(v+\frac{1}{2}\right)-\omega_{e}x_{e}{\left(v+\frac{1}{2}\right)}^{2}\;.$$

A practical procedure we follow is to compute data for the potential energy curves for a set of values $R_{i}$, which span the region of *R*-values such that one obtains a realistic energy spectrum for low-lying nuclear vibrations of the molecule, v=0,1,2,3. A spline fit through the data allows to determine $R_{e}$, and the value of the minimum potential energy $T_{e}$.

It is convenient to fit these data to a Morse potential,

(2) $$V\left(R\right)=T_{e}+D\;{\left(1-\exp\left[-\beta \left(R-R_{e}\right)\right]\right)}^{2}\;.$$

The two additional constants *D* and β can then be found from the fit. Here, *D* is just a parameter (not necessarily close to the dissociation energy), as the purpose of fitting to a Morse potential is to obtain a wider range in *R* to accommodate the tails of the vibrational states when solving the nuclear Schrödinger equation. This procedure avoids the computation at a larger grid of values $R_{i}$. Alternatively, one can carry out direct fits of the original data to the Morse potential Equation (2) to find the four parameters.

Fitting the vibrational spectrum for v=0,1,2,3 to Equation (1) allows one to find the parameters $\omega_{e}$ and $\omega_{e}x_{e}$, or a more complete set including $\omega_{e}y_{e}$ in case one uses not a Morse potential but a more general fit of the calculated potential energy points at the given set $\left\{R_{i}\right\}$. For the Morse potential, the values of $\omega_{e}$ and $\omega_{e}x_{e}$ are related directly to the potential parameters.

One can also deduce the parameters from the experimentally determined values of the spectroscopic constants. In atomic units, one has

(3) $$D=\frac{\omega_{e}^{2}}{4\omega_{e}x_{e}}\;\beta =\sqrt{\frac{M}{2D}}\omega_{e}\;,$$

where *M* is the reduced mass of the diatomic molecule.

In terms of expected accuracy for the excitation energies, the goal is to be within $100\;{\mathrm{cm}}^{-1}$ of the experimentally deduced values. Is this percent level (or better) goal achievable? The EOM-CC3 method is claimed to be good to 40meV level (≈$300\;{\mathrm{cm}}^{-1}$) [21], in general. The reason why we can expect to do better than that for our cases is that the excitations are predominantly of the single-electron type. In the case of MgF, it is shown that the method, when coupled with the augmented correlation consistent basis sets, can deliver per mille accuracy for transition energies [14].

The results were obtained on multi-core workstations (e.g., Apple MacBook Pro with 8 cores, but during most of the computations, the CPU was loaded only to the 40% level). Excited-state calculation using the EOM-CC3 method for a single root took of the order of 10–12 h with the aug-cc-pV5Z-PP basis and about half the time for cc-pV5Z-PP. Shorter computing times were found while using the Linux implementation of Psi4 (version 1.9.1).

## 3. Results

### 3.1. SrF($X^{\;2}\Sigma^{+}\to B^{\;2}\Sigma^{+}$)

**Figure 1:**
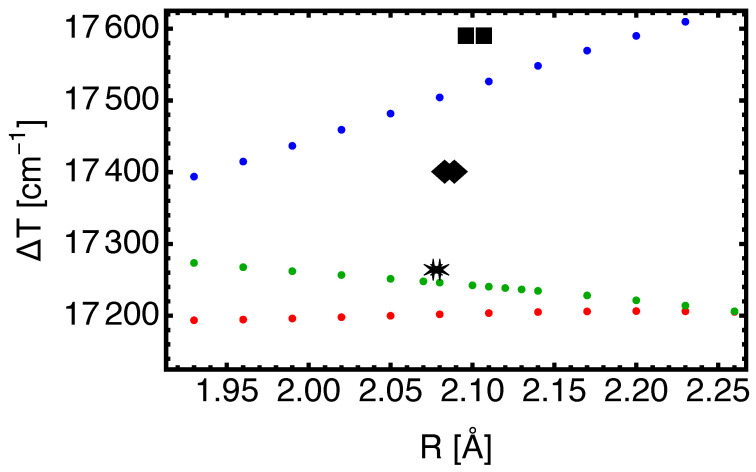
Vertical excitation energies ΔT in ${\mathrm{cm}}^{-1}$ as a function of *R* (in Å) for the electronic transition SrF$\left(X^{\;2}\Sigma^{+}\to B^{\;2}\Sigma^{+}\right)$. The blue, red, and green dots represent the EOM-CC3 results obtained with the aug-cc-pVnZ-PP family with n = 3, 4, 5, respectively. For R=2.08 Å and n=4,5, the vertical excitation energies correspond to $T_{e}$ according to the present work. Stars: experimental value extracted from the analysis of rotational and vibrational (v=0,1,2) excitations [23]; the symbol location indicates the values of $R_{e}$ for both states. Other theoretical values for $T_{e}$: squares are the CAS-SCF/MRCI values (Ref. [27]), and diamonds are X2C-FSCC (Ref. [3]).

As a first case, we look into an excitation of SrF for which the equilibrium distances of the ground and excited states are very close to each other, namely, R=2.075 and R=2.08 Å respectively. Vertical excitation energies are shown in Figure 1 as a function of *R* over a range of values that are needed to determine the basic spectroscopic parameters. The variation in the excitation energies with *R* is large for the aug-cc-pVnZ basis with n=3, i.e., TZ, but less for the QZ (n=4) and 5Z (n=5) calculations.

The obtained values of $R_{e},\Delta T_{e},\omega_{e},\omega_{e}x_{e}$ for both states are compared in Table 1 with values derived from experiments, and with the Fock space (FS) CC calculation of Ref. [3]. The variation of $\Delta T_{e}$ with the basis set quality parameter n=3,4,5 is not systematic, and the validity of extrapolation to the complete basis set (CBS) limit may be questioned, and is therefore omitted.

The spectroscopic parameters $\omega_{e}$ and $\omega_{e}x_{e}$ were determined in the following way: the potential energy data for both states were obtained on a grid of 16 equidistant *R* values spanning 1.93≤R≤2.26; these data were fitted to a local spline using the Interpolation function in Mathematica; the minimum position $R_{e}$ and energy $T_{e}$ were determined using FindMinimum; and after the subtraction of $T_{e}$, the data were then fitted to either a Morse potential, or to a fourth-order polynomial including orders 2, 3, and 4, and centered on the determined value of $R_{e}$. Using the Morse and polynomial fits, the Schrödinger equation for the nuclear motion was solved with NDEigensystem in Mathematica using the reduced mass for ^88^Sr^19^F, which is the most abundant isotope, and which was selected in experiments [22,23,24].

**Table 1:** Spectroscopic data for the $X^{\;2}\Sigma^{+}$ and $B^{\;2}\Sigma^{+}$ states of ^88^Sr^19^F. The values of $R_{e}$ are given in Å, while all energies are given in ${\mathrm{cm}}^{-1}$. Data extracted from the present EOM-CC3 calculations using the aug-cc-pVnZ-PP basis sets [17] with effective core potential ECP28MDF [16] for Sr and aug-cc-pVnZ for F are shown for n=3,4,5.

	$X^{\;2}\Sigma^{+}$			$B^{\;2}\Sigma^{+}$			
	$R_{e}$	$\omega_{e}$	$\omega_{e}x_{e}$	$R_{e}$	$\omega_{e}$	$\omega_{e}x_{e}$	$\Delta T_{e}$
Expt [23,25]	2.076	501	2.27	2.080	496	2.34	17,264
X2C-FSCC [3]	2.083	500	2.45	2.089	492	2.16	17,405
EOM-CC3(5Z)	2.079	495	2.1	2.081	493	2.1	17,246
EOM-CC3(4Z)	2.075	507	2.2	2.074	507	2.3	17,202
EOM-CC3(3Z)	2.099	510	2.3	2.093	516	2.4	17,516

For the 5Z basis, the present results work very well with the excitation energy $\Delta T_{e}$ obtained within $20\;{\mathrm{cm}}^{-1}$, which is an excellent result. The focus on the excitation to the SrF($B^{\;2}\Sigma^{+}$) state in this section is motivated by the case of BaF, for which one can argue that effective and true excitation energies may differ by over $100\;{\mathrm{cm}}^{-1}$ [26]. The problem of state mixing of one excited state with a vibrationally excited neighboring state becomes a problem for BaF due to the closeness of the ${A^{\prime }}^{\;2}\Delta$ and $A^{\;2}\Pi$ potential energy curves. The potential energy curves for the sequence of CaF, SrF, and BaF molecules are shown in Figure 1 of Ref. [3]. For SrF (in contrast to BaF), there should not be a problem when comparing effective experimental and theoretical parameters for the $B^{\;2}\Sigma^{+}$ state. 

A comment on the comparison with the calculations of Ref. [3] is perhaps in order. The scope of that work, of course, is substantially wider: the relativistic framework with an effective Hamiltonian for the molecular mean-field approach is reduced to a two-component formalism (hence, it is designated as the X2C-FSCC method). It includes part of the Breit interaction, and a basis set was constructed in a careful way with the doubly augmented d-aug-pVQZ relativistic basis of Dyall [28] expanded manually. This effort was required due to the treatment of the all-electron problem, and resulted in accurate spin–orbit splittings (which is not relevant for the $B^{\;2}\Sigma^{+}$ state). In contrast, the present work describes the relativistic effects by a pseudopotential for the inner 28 electrons of Sr (ECP28 based on multi-configuration Dirac–Fock and Breit interaction, cf. Ref. [16]), and uses a correlation-consistent basis set sequence designed for the atom [17]. Within the EOM-CC3 method, this much simpler approach yields an improved excitation energy, and somewhat better values for the equilibrium distances.

### 3.2. BaF($X^{\;2}\Sigma^{+}\to {A^{\prime }}^{\;2}\Delta$, $X^{\;2}\Sigma^{+}\to A^{\;2}\Pi$, $X^{\;2}\Sigma^{+}\to B^{\;2}\Sigma^{+}$)

In order to compare ECP-based calculations which are non-relativistic in nature, except for taking into account the contributions from core electrons of the heavy atom via the pseudopotential, we need to look at the spectroscopic results where the spin–orbit splittings are removed. A detailed analysis of BaF low-lying electronic excitations led to the discovery of the lowest excitation, namely, the ${A^{\prime }}^{\;2}\Delta$ doublet [29]. The paper gives the excitation energies $\Delta T_{e}$ and spectroscopic parameters for the levels considered here, but the experiment naturally includes the spin–orbit effect associated with $J=\frac{3}{2},\frac{5}{2}$ for ${A^{\prime }}^{\;2}\Delta$, and $J=\frac{1}{2},\frac{3}{2}$ for $A^{\;2}\Pi$. A simple approach to obtain reference values for comparison with the present results is to average the spin–orbit splittings. Those values are within $1\;{\mathrm{cm}}^{-1}$ of those shown in Table 2, which are taken from the later analysis of an expanded data set.

In a follow-up paper [30], a detailed analysis was reported for the vibration–rotation bands v=0,1,2, and effective constants were determined. These were derived from a model and resulted in the determination of spin–orbit coupling constants. The study was then complemented by a deperturbation approach [26], based on the notion that the excited states are predominantly 5d states. Another set of effective excitation energies was determined. The deperturbation concerns the fact that the v=2 vibrational excitation of the ${A^{\prime }}^{\;2}\Delta$ state becomes close to the v=0$A^{\;2}\Pi$ state. Due to spin–orbit couplings, the $B^{\;2}\Sigma^{+}$ state is strongly affected, and the excitation energies $\Delta T_{e}$ derived from the experimental data may not be exactly the quantities expected from the theoretical potential energy curves.

**Table 2:** Experimentally derived effective spectroscopic parameters for the low-lying electronic states of BaF. The values in columns 2 to 5 are taken from Table III in Ref. [31] (with the least certain digits truncated), while the values of $R_{e}$ are taken from Table III in Ref. [3]. All energies are given in ${\mathrm{cm}}^{-1}$.

State	$\Delta T_{e}$	$\omega_{e}$	$\omega_{e}x_{e}$	$10^{2}\omega_{e}y_{e}$	$R_{e}$[Å]
$X^{\;2}\Sigma^{+}$	0	469.416	1.837	0.33	2.1593
${A^{\prime }}^{\;2}\Delta$	10,940.3	437.4	1.83		-
$A^{\;2}\Pi$	11,962.2	437.9	1.85		2.183
$B^{\;2}\Sigma^{+}$	14,062.5	424.8	1.85	0.39	2.208

More complete measurements analyzing higher vibrational states are reported in Ref. [31], and the spectroscopic parameters are given in Table 2 as reference values. Recently, measurements and analysis were performed not only for the most abundant isotope ^138^Ba^19^F but also for ^136^Ba^19^F [32].

The deperturbed values of $\Delta T_{e}$ given in Table 3 of Ref. [26] agree with the simple averaging procedure shown in Table 3 for the ${A^{\prime }}^{\;2}\Delta$ state, are about $17\;{\mathrm{cm}}^{-1}$ higher for the $A^{\;2}\Pi$ state, and are about $120\;{\mathrm{cm}}^{-1}$ lower for the $B^{\;2}\Sigma^{+}$ state ($T_{\Sigma }$ = 13,944.5). This should be kept in mind when looking at the comparison with theory.

On the theoretical side, we note that the relativistic FSCC method of Ref. [3] gives higher excitation energies for the properly coupled states with some overestimations at the $150\;{\mathrm{cm}}^{-1}$ level for ${A^{\prime }}^{\;2}\Delta$ and $A^{\;2}\Pi$. The spin–orbit splittings are obtained to reasonable accuracy. The same cannot be said for the CASSCF+MRCI method with spin–orbit coupling calculated with perturbation theory [33], and our own experience while using Molpro (version 2023.2) [34] in an all-electron approach was similar in this respect.

How useful are calculated vertical excitation energies ΔT as approximations for $\Delta T_{e}$? For the $A^{\;2}\Pi$ and ${A^{\prime }}^{\;2}\Delta$ states, this turns out to work but less so for the $B^{\;2}\Sigma^{+}$ state due to the variation in ΔT(R) as will be shown below. Our EOM-CC3 results for R=2.16 Å, with an augmented basis for both Ba and F, are shown in Table 3. For the excitation to the $B^{\;2}\Sigma^{+}$ state, the difference between $\Delta T_{e}$ and ΔT is appreciable.

Overall, the results are quite good but with substantial room for improvement for excitation to the ${A^{\prime }}^{\;2}\Delta$ state due to the overestimation by about $230\;{\mathrm{cm}}^{-1}$. For the $B^{\;2}\Sigma^{+}$ state, the result for $\Delta T_{e}$ supports the notion that theory should be compared to the value from the deperturbation analysis [26], and not with the effective constant given in Refs. [29,31].

**Figure 2:**
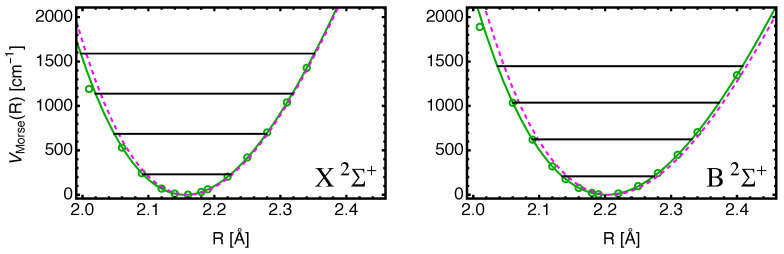
Morse potentials derived from the calculated data points (green circles) are shown as solid green curves for the states BaF($X^{\;2}\Sigma^{+}$) on the left, and BaF($B^{\;2}\Sigma^{+}$) on the right. The magenta dashed curves show experimentally determined Morse potentials using Equation (3) and values from Ref. [31]. The black vertical lines indicate the calculated vibrational energy levels for v=0,1,2,3. The data points are for the aug-cc-pV5Z-PP/aug-cc-PV5Z basis set combination. The fits omit the data points for R=2.01 Å, (cf. text).

In order to assess the quality of the EOM-CC3 results, we show in Figure 2 the calculated potential energies as a function of separation *R* with $T_{e}$ subtracted for the $X^{\;2}\Sigma^{+}$ and $B^{\;2}\Sigma^{+}$ states. Also shown are fits to the data using the Morse potential Equation (2). The data points for R=2.01 Å are removed from the fit, and disagree with the Morse shape for both states. The reason for this deviation may be associated with basis set problems (superposition error) for small values of *R*. The dashed magenta curves are the potentials derived from the experimental parameters $R_{e},\omega_{e},\omega_{e}x_{e}$ shown in Table 2 using Equation (3).

The agreement is not perfect: the results from the *R* values to the left of the minima may be affected by the mentioned problem for the first data point, particularly in the case of the $X^{\;2}\Sigma^{+}$ state. The values for $\omega_{e}x_{e}$ depend somewhat on the choice of data points used for the fits, and, thus, their values shown in Table 4 are, at best, estimates. The values for $R_{e}$ should be accurate as stated, and those for $\omega_{e}$ are deemed to be accurate to two–three digits.

In Table 5, we present the results from different CC methods and two basis set combinations for the vertical excitation energies at R=2.16 Å. We focus on the comparison of basis sets aug-cc-pV5Z-PP and cc-pV5Z-PP for the barium atom combined with aug-cc-pV5Z for fluorine since the latter combination allowed us to apply state-specific CC methods in addition to the EOM approach.

We can summarize the data as follows: the best calculations are given in the second row for EOM-CC3 with augmentation on both atoms. Removing augmentation on Ba results in literally no change for the excitation to $A^{\;2}\Pi$ and $B^{\;2}\Sigma^{+}$ states, and a small increase for the ${A^{\prime }}^{\;2}\Delta$ state. Interestingly, the excitation energies from the Δ-CC3 method are higher than the EOM-CC3 results with the same basis on the order of 40–50 cm^−1^. The EOM-CCSD calculations yield systematically higher excitation energies, and are, thus, deemed less useful. The Δ-CCSD results, on the other hand are lower for the $B^{\;2}\Sigma^{+}$ state. The Δ-CCSD(T) method gives results close to those from the Δ-CC3 method. In Table A1 in the Appendix A, the energies are given for completeness. One can observe that the actual energies for different methods disagree much more than the excitation energies.

Concerning the state-specific Δ-CC results for excited states, which are obtained from higher roots than the ground state, we make the following observations. The Δ-SCF solutions, which are required as an orbital basis to perform the CC steps, are easy to find in $C_{2v}$ or $C_{1}$ symmetry for the $A^{\;2}\Pi$ and $B^{\;2}\Sigma^{+}$ states, by replacing the highest occupied with nearby lowest unoccupied natural orbitals (HONO vs. LUNO+j) with j=0,1 using Psi4 terminology. For the ${A^{\prime }}^{\;2}\Delta$ state, we used $C_{s}$ symmetry and used the LUNO+4 to replace the HONO to obtain the Δ-SCF Slater determinant. The CCSD(T) calculation generates the CCSD energy as an intermediate result, while the Δ-CC3 calculations are performed separately. The convergence properties of the CC correlation energy calculations are similar to ground-state calculations (on the order of 20 iterations) when working in $C_{s}$ or $C_{2v}$ symmetry.

**Figure 3:**
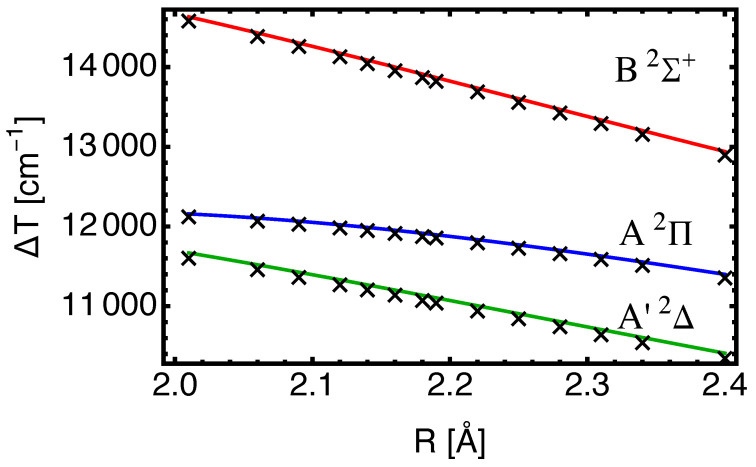
EOM-CC3 vertical excitation energies ΔT in ${\mathrm{cm}}^{-1}$ as a function of *R* (in Å) for the electronic transitions $X^{\;2}\Sigma^{+}\to {A^{\prime }}^{\;2}\Delta$ (green), $X^{\;2}\Sigma^{+}\to A^{\;2}\Pi$ (blue), and $X^{\;2}\Sigma^{+}\to B^{\;2}\Sigma^{+}$ (red). The curves represent spline fits to data obtained with the basis cc-pV5Z-PP for barium and aug-cc-pV5Z for fluorine. The crosses are data points obtained with both basis sets augmented, which lowers the values for the excitation energies by up to $30\;{\mathrm{cm}}^{-1}$.

In Figure 3, the excitation energies from EOM-CC3 with both basis sets are shown as a function of internuclear separation *R*. The dependence on *R* emerges from the structure calculation and is connected with the fact that the potential energy surfaces have different shapes and minima at state-dependent values of $R_{e}$ as shown in Figure 2. The crosses show the data with augmentation on both atoms and are calculated on a fine grid of *R*-values (cf. Table A1). The curves show the strong dependence of ΔT vs. *R* for the excitation to $B^{\;2}\Sigma^{+}$, which is responsible for the difference between ΔT and $\Delta T_{e}$ for this state (cf. Table 2). The basis set combination which lacks augmentation on Ba gives nevertheless results of good quality, and may be preferred for more complicated situations (such as BaF within a cryogenic Ne matrix).

The $X^{\;2}\Sigma^{+}$ ground-state energies for R=2.16 Å which are shown in the Appendix A in Table A1 depend strongly on which CC method is used, and also show variation with the level of basis (aug/aug vs. cc/aug for Ba/F). Nevertheless, the vertical excitation energies from the Δ-CC methods differ from EOM-CC3 on the $100\;{\mathrm{cm}}^{-1}$ scale for Δ-CCSD and $30\;{\mathrm{cm}}^{-1}$ for Δ-CCSD(T).

At the level of EOM-CC3, we find that only the ${A^{\prime }}^{\;2}\Delta$ state excitation energy changes noticeably (it comes out higher by about $30\;{\mathrm{cm}}^{-1}$, which is on top of an ≈230 cm^−1^ overestimate). For the sake of computational economy, one may recommend this approach. Neglecting the triples (CCSD over CCSD(T) or CC3) leads to ≈100 cm^−1^ differences in the excitation energies.

In order to assess the quality of the results with respect to basis size, we provide in Table 6 results for the vertical excitation energies at R=2.16 Åas obtained with the EOM-CC3 method and basis set levels n=3,4,5. The main purpose is to demonstrate that the results for excitation to $A^{\;2}\Pi$ and $B^{\;2}\Sigma^{+}$ do not change significantly between n = 4 and n = 5 but that the ${A^{\prime }}^{\;2}\Delta$ energies are not converged yet at the 5Z level. This conclusion is supported by results for both the fully augmented basis sets (augmentation on Ba/F), and the calculations with augmentation only on the F atom.

For the problematic state, i.e., ${A^{\prime }}^{\;2}\Delta$, substantial energy decreases occur for both steps, i.e., for n=3→4, and n=4→5. Extrapolation to the CBS limit falls below the experimental value of $\Delta T_{e}\approx$ 10,940 $\;{\mathrm{cm}}^{-1}$, i.e., potentially resulting in too low a value for ΔT by $100\;{\mathrm{cm}}^{-1}$. Thus, extrapolation has to be taken with a grain of salt for excited states. For the other two states, the step n=4→5 counteracts the decrease in ΔT somewhat, and the changes are much smaller.

One can make the following observations on the basis of these results: the differences between n = 4 and n = 5 results are small for the $B^{\;2}\Sigma^{+}$ state, and the results practically do not depend on whether the Ba atom basis is augmented or not. For the $A^{\;2}\Pi$ state, the differences between n = 4 and n = 5 are about $40\;{\mathrm{cm}}^{-1}$, and also do not really change, whether augmentation on Ba is included or not. For the ${A^{\prime }}^{\;2}\Delta$, state augmentation on Ba changes the results for n = 3 and n = 4 but less so for n = 5.

## 4. Conclusions

EOM-CC3 calculations for electronic excitations of SrF and BaF molecules are reported on the basis of treating these molecules as 19-electron systems, while representing the inner cores of Sr and Ba with pseudopotentials, which effectively removes 28 or 46 electrons for the two heavy atoms.

For SrF, only the $X^{\;2}\Sigma^{+}\to B^{\;2}\Sigma^{+}$ transition is investigated. The difference between the equilibrium nuclear separations $R_{e}$ for the two states is small. It is shown how the vertical excitation energy changes with separation *R* as a function of basis set parameter n. For the QZ and 5Z basis (aug-cc-pVnZ-PP/aug-cc-pVnZ for Sr/F), this variation is much less pronounced than for TZ. The resulting value for $\Delta T_{e}$ at the 5Z basis level is $20\;{\mathrm{cm}}^{-1}$ below the experimentally determined value, which is substantially better than other calculations. The variation of ΔT for fixed *R* as a function of basis set quality parameter n=3,4,5 is not systematic, and therefore the extrapolation to the complete basis set limit for $\Delta T_{e}$ appears to be less valid than for the ground-state energy.

For the BaF molecule, calculations are carried out for excitations to three closely related states, namely, ${A^{\prime }}^{\;2}\Delta$, $A^{\;2}\Pi$, and $B^{\;2}\Sigma^{+}$ using basis sets at the 5Z level. The $\Delta T_{e}$ values obtained for the ${A^{\prime }}^{\;2}\Delta$ state overestimate the experimentally derived value by about $230\;{\mathrm{cm}}^{-1}$, while the results for $A^{\;2}\Pi$ and for $B^{\;2}\Sigma^{+}$ agree to within $30\;{\mathrm{cm}}^{-1}$ of the spectroscopic value obtained from a deperturbation analysis [26]. A study of the vertical excitation energies as a function of basis parameter n reveals the lack of convergence even at n=5 for the ${A^{\prime }}^{\;2}\Delta$ state, while showing reasonable convergence behavior for the two other states.

The present calculation for the $B^{\;2}\Sigma^{+}$ excitation energy based on scalar-relativistic Born–Oppenheimer potential energy curves agrees better with the deperturbation analysis [26] than with the effective parameters derived directly from the experimental data [31]. The latter parameters are, however, explained properly in a full relativistic calculation, such as in Refs. [3,4].

## Figures and Tables

**Table 3:** The present EOM-CC3 results for ΔT (R=2.16 Å) and $\Delta T_{e}$, using the aug-cc-pV5Z-PP basis set [17] with effective core potential ECP46MDF [16] for Ba and aug-cc-pV5Z basis for F, are shown in columns 2 and 3. The deperturbation results of Ref. [26] are given in column 4, while the effective spectroscopic values from Ref. [31] are given in column 5. Note that the deperturbed $T_{e}$ value for the $B^{\;2}\Sigma^{+}$ state is lower by about $120\;{\mathrm{cm}}^{-1}$ compared to the effective value given in Ref. [31].

State	ΔT	$\Delta T_{e}$	Ref. [26]	Ref. [31]
${A^{\prime }}^{\;2}\Delta$	11,178	11,168	10,938.9	10,940.3
$A^{\;2}\Pi$	11,952	11,947	11,979.6	11,962.2
$B^{\;2}\Sigma^{+}$	13,995	13,927	13,944.5	14,062.5

**Table 4:** Spectroscopic parameters as obtained from EOM-CC3 calculations using the cc-pV5Z-PP basis set [17] for Ba and the aug-cc-pV5Z basis set for F are shown in the upper rows, while the aug-cc-pV5Z-PP/aug-cc-pV5Z basis set results are shown in the lower rows for each state. The equilibrium separations $R_{e}$ are given in Å, while $\omega_{e}$ and $\omega_{e}x_{e}$ are given in ${\mathrm{cm}}^{-1}$.

State	$R_{e}$	$\omega_{e}$	$\omega_{e}x_{e}$
$X^{\;2}\Sigma^{+}$	2.153	456	1.6
aug/aug:	2.155	459	1.5
${A^{\prime }}^{\;2}\Delta$	2.189	427	1.4
aug/aug:	2.188	430	1.7
$A^{\;2}\Pi$	2.173	428	1.4
aug/aug:	2.174	432	1.7
$B^{\;2}\Sigma^{+}$	2.201	417	1.3
aug/aug:	2.202	419	1.6

**Table 5:** Vertical excitation energies ΔT (in ${\mathrm{cm}}^{-1}$) from the $X^{\;2}\Sigma^{+}$ ground state at R=2.16 Åas calculated with different methods and two basis sets. The results for the augmented basis sets on both Ba and F are marked with ^†^.

Method	${A^{\prime }}^{\;2}\Delta$	$A^{\;2}\Pi$	$B^{\;2}\Sigma^{+}$
EOM-CC3	11,202	11,953	14,000
EOM-CC3 ^†^	11,178	11,952	13,995
Δ-CC3	11,212	11,992	14,051
EOM-CCSD	11,191	12,030	14,164
EOM-CCSD ^†^	11,164	12,029	14,157
Δ-CCSD	11,177	11,821	13,932
Δ-CCSD(T)	11,185	11,961	14,027

**Table 6:** Vertical excitation energies ΔT (in ${\mathrm{cm}}^{-1}$) from the $X^{\;2}\Sigma^{+}$ ground state at R=2.16 Å, as calculated with the EOM-CC3 method and basis sets with n = 3, 4, 5 without augmentation on the Ba atom in the top half and with augmentation on both Ba/F in the bottom half (marked by ^†^).

Basis	${A^{\prime }}^{\;2}\Delta$	$A^{\;2}\Pi$	$B^{\;2}\Sigma^{+}$
n = 3	11,885	11,975	14,058
n = 4	11,765	11,911	13,994
n = 5	11,202	11,953	14,000
CBS	10,869	11,978	14,004
n = 3 ^†^	11,822	11,968	14,020
n = 4 ^†^	11,715	11,912	13,974
n = 5 ^†^	11,178	11,952	13,995
CBS ^†^	10,860	11,976	14,008

## Data Availability

Data are contained within the article.

## Appendix A

**Table A1:** The top eight rows give energies (in Hartree) from EOM-CC3 calculations using the cc-pV5Z-PP basis set [17] for Ba and the aug-cc-pV5Z basis set for F. The scale of the correlation energy is 0.6 Hartree. The EOM-CC3 results are in the bottom part of the table, and the EOM-CCSD results marked with ^†^ are obtained with the aug-cc-pV5Z-PP basis for Ba and the aug-cc-pV5Z basis set for F. The excitation energies from these results are represented by open circles in Figure 3. The state-specific Δ-CC results are obtained with the cc-pV5Z-PP basis for Ba and the aug-cc-pV5Z basis set for F, and were obtained with symmetry set to $C_{1}$.

Method	*R* [Å]	$X^{\;2}\Sigma^{+}$	${A^{\prime }}^{\;2}\Delta$	$A^{\;2}\Pi$	$B^{\;2}\Sigma^{+}$
EOM-CC3	2.01	−125.27549126	−125.22232710	−125.22009727	−125.20882965
	2.06	−125.27873089	−125.22624470	−125.22357258	−125.21298780
	2.11	−125.28070218	−125.22892852	−125.22585778	−125.21592208
	2.16	−125.28116764	−125.23012756	−125.22670690	−125.21737647
	2.21	−125.28049152	−125.23019824	−125.22647458	−125.21770545
	2.26	−125.27885127	−125.22931269	−125.22532878	−125.21707479
	2.31	−125.27641989	−125.22763820	−125.22343428	−125.21565498
	2.36	−125.27334842	−125.22532102	−125.22093362	−125.21358932
EOM-CCSD	2.16	−125.25688898	−125.20589866	−125.20207560	−125.19235173
EOM-CCSD ^†^	2.16	-125.25729908	-125.20643000	-125.20249267	-125.19279287
Δ-CCSD	2.16	−125.25688897	−125.20596253	−125.20302866	−125.19340808
Δ-CCSD(T)	2.16	−125.27876575	−125.22780103	−125.22426517	−125.21485159
Δ-CC3	2.16	−125.28116806	−125.23008276	−125.22652706	−125.21714501
EOM-CC3 ^†^	2.01	−125.27619337	−125.22314957	−125.22078430	−125.20959123
	2.06	−125.27919971	−125.22682037	−125.22403798	−125.21348092
	2.09	−125.28051516	−125.22856143	−125.22553502	−125.21536980
	2.12	−125.28130166	−125.22978244	−125.22652966	−125.21674161
	2.14	−125.28155940	−125.23033380	−125.22693976	−125.21739462
	2.16	−125.28161379	−125.23068403	−125.22715668	−125.21784780
	2.18	−125.28147360	−125.23084251	−125.22718908	−125.21810835
	2.19	−125.28133651	−125.23085492	−125.22714142	−125.21817269
	2.22	−125.28069044	−125.23065988	−125.22677682	−125.21813306
	2.25	−125.27970333	−125.23012609	−125.22608895	−125.21775394
	2.28	−125.27842499	−125.22930182	−125.22512556	−125.21708388
	2.31	−125.27688344	−125.22821412	−125.22391279	−125.21614988
	2.34	−125.27511229	−125.22689580	−125.22248262	−125.21498428
	2.40	−125.27098835	−125.22367141	−125.21907086	−125.21206175
